# The choroid plexus may be an underestimated site of tumor invasion to the brain: an in vitro study using neuroblastoma cell lines

**DOI:** 10.1186/s12935-015-0257-2

**Published:** 2015-10-24

**Authors:** Elodie Vandenhaute, Carolin Stump-Guthier, María Lasierra Losada, Tobias Tenenbaum, Henriette Rudolph, Hiroshi Ishikawa, Christian Schwerk, Horst Schroten, Matthias Dürken, Martin März, Michael Karremann

**Affiliations:** 1 Laboratoire de la Barrière Hémato-Encéphalique (LBHE), EA 2465, Université d’Artois (UArtois), Lens, France; 2 Department of Pediatric and Adolescent Medicine, University Medical Center Mannheim, Medical Faculty Mannheim, Heidelberg University, Mannheim, Germany; 3 Institute of Experimental Medicine I, Nikolaus-Fiebiger Center for Molecular Medicine, Friedrich-Alexander University of Erlangen-Nürnberg, Erlangen, Germany; 4 Department of NDU Life Sciences, School of Life Dentistry, The Nippon Dental University, Tokyo, Japan

**Keywords:** Blood-cerebrospinal fluid barrier, Brain metastases, Choroid plexus, In vitro model, Neuroblastoma, Cancer cell transmigration

## Abstract

**Background:**

The central nervous system (CNS) is protected by several barriers, including the blood–brain (BBB) and blood-cerebrospinal fluid (BCSFB) barriers. Understanding how cancer cells circumvent these protective barriers to invade the CNS is of crucial interest, since brain metastasis during cancer is often a fatal event in both children and adults. However, whereas much effort has been invested in elucidating the process of tumor cell transmigration across the BBB, the role of the BCSFB might still be underestimated considering the significant number of meningeal cancer involvement. Our work aimed to investigate the transmigration of neuroblastoma cells across the BCSFB in vitro.

**Methods:**

We used an inverted model of the human BCSFB presenting proper restrictive features including adequate expression of tight-junction proteins, low permeability to integrity markers, and high trans-epithelial electrical resistance. Two different human neuroblastoma cell lines (SH-SY5Y and IMR-32) were used to study the transmigration process by fluorescent microscopy analysis.

**Results:**

The results show that neuroblastoma cells are able to actively cross the tight human in vitro BCSFB model within 24 h. The presence and transmigration of neuroblastoma cancer cells did not affect the barrier integrity within the duration of the experiment.

**Conclusions:**

In conclusion, we presume that the choroid plexus might be an underestimated site of CNS invasion, since neuroblastoma cell lines are able to actively cross a choroid plexus epithelial cell layer. Further studies are warranted to elucidate the molecular mechanisms of tumor cell transmigration in vitro and in vivo.

**Electronic supplementary material:**

The online version of this article (doi:10.1186/s12935-015-0257-2) contains supplementary material, which is available to authorized users.

## Background

Neuroblastoma is a common extracranial pediatric solid tumor, arising from the sympathetic nervous system. At the time of diagnosis, more than half of the patients already exhibit metastases in distant organs, including lymph nodes, bone marrow, bone and other organs. Even if central nervous system (CNS) involvement is rarely described in neuroblastoma and restricted to tumor relapse, its frequency is increasing and its prognosis is dismal [1]. Efforts have been made to develop treatments for CNS metastases, but they are generally ineffective in preventing progression and subsequent death [2]. The pattern of spread is mostly believed to occur via the hematogenous route, implying that circulating cancer cells reach the CNS after crossing the interfaces existing between the blood and the brain. Among these, the blood–brain barrier (BBB) lies in the restrictive microvascular walls, whereas the blood-cerebrospinal fluid barrier (BCSFB) is formed by the choroid plexus epithelial cells [3]. Many studies have focused on the migration of cancer cells through the blood–brain barrier, whereas the BCSFB is underestimated as a potential route to reach the CNS. In the frame of neuroblastoma, CNS metastatic lesions are indeed not only parenchymal, but intraventricular lesions and meningeosis have also been described [4, 5]. Our aim was to elucidate the migratory potential of neuroblastoma cells to cross the intact BCSFB. For this purpose, we studied the transmigration of two neuroblastoma cell lines—IMR32 and SH-SY5Y cells—through a well-established human in vitro BCSFB model. Our results show that these neuroblastoma cell lines could actively cross the choroid plexus epithelium within 24 h to a similar extent, and that the interactions with cancer cells did not cause a breakdown of the cellular barrier.

## Results

### IMR32 and SH-SY5Y cells can cross the intact human in vitro BCSFB within 24 h and do not affect its overall integrity

**Fig. 1:**
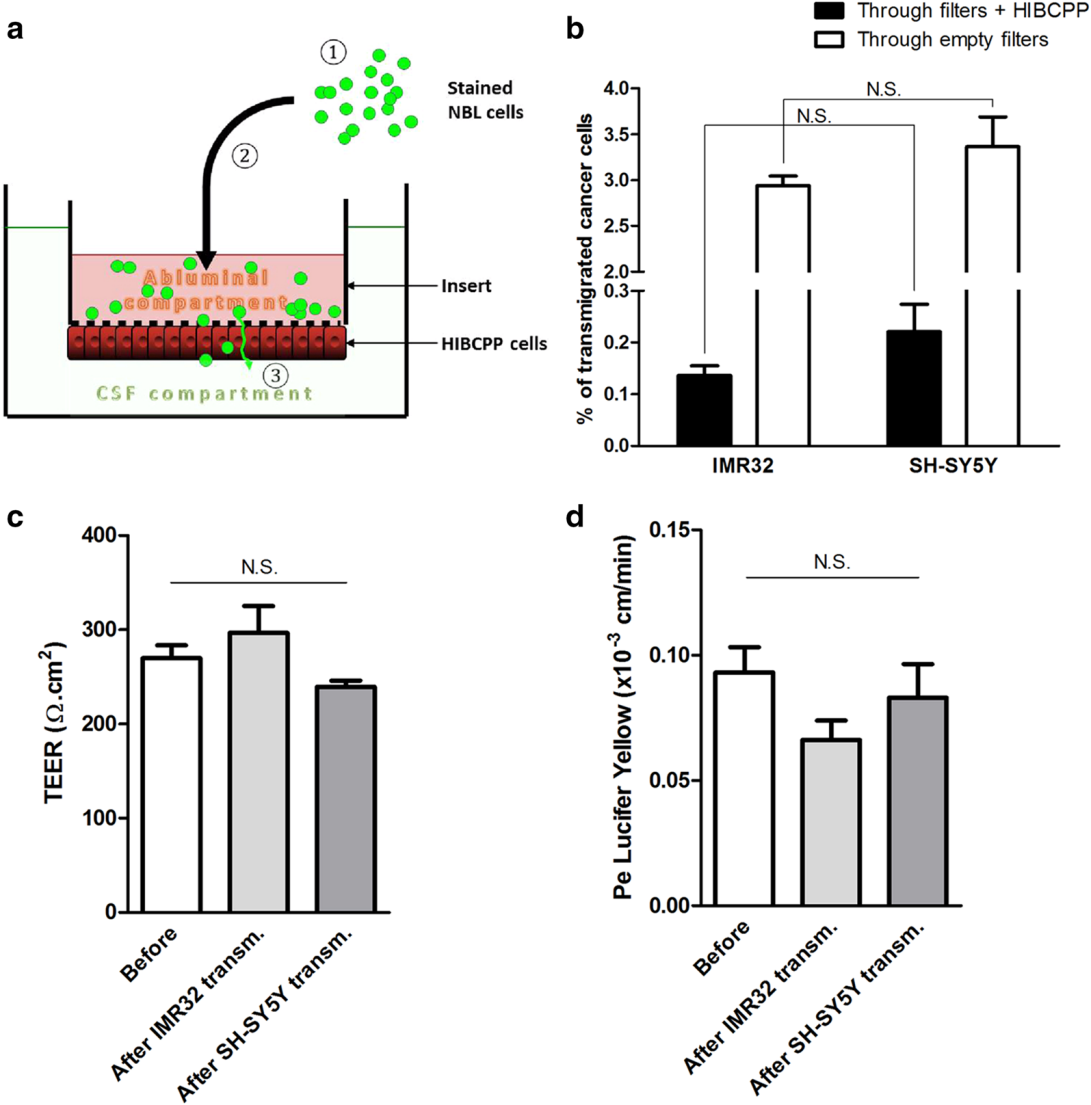
The blood-cerebrospinal fluid barrier (BCSFB) in vitro model (**a**) used for transmigration experiments (**b**) and effect of the transmigration process on the barrier function (**c**, **d**). **a** The BCSFB model is based on the culture of human choroid plexus papilloma cells (HIBCPP cells) on the lower surface of the inserts, so that the upper side mimics the blood compartment and the lower one the CSF compartment. For the transmigration experiment, neuroblastoma cells were dissociated and intracellularly stained using BCECF-AM (*1*), before being seeded in the upper compartment (*2*). At the end of the experiment (*3*), the number of transmigrated cells was assessed by fluorescence measurements, and the barrier integrity was evaluated by measuring the trans-epithelial electrical resistance (TEER) and the permeability of the barrier to Lucifer Yellow (LY, integrity marker). **b** Percentage of transmigrated neuroblastoma cells (having reached the lower compartments after crossing filters alone, or filters + HIBCPP layer), as assessed by fluorescence measurements. **c** TEER values (in Ω cm^2^) before and after the transmigration of IMR32 and SH-SY5Y cells. **d** Permeability coefficient (Pe, in cm/min) for LY measured on control filters and after the transmigration of IMR32 and SH-SY5Y cells. *N.S.* non significant

The transmigration of neuroblastoma cell lines was studied through the human BCSFB in vitro (Fig. 1), which consists of polarized HIBCPP cells [6]. For convenience and as already described [6], we used the inverted model where HIBCPP cells are cultivated on the bottom of the inserts, so that the blood side corresponds to the upper compartment (Fig. 1a). In basal conditions, choroid plexus epithelial cell cultures exhibited high electrical resistance (Fig. 1c, 269.9 ± 13.5 Ω cm^2^) and low permeability to Lucifer Yellow (LY) integrity marker (Fig. 1d, 0.09 ± 0.01 × 10^−3^ cm/min), demonstrating the formation of a restrictive barrier. Neuroblastoma cells were harvested, dissociated and incubated in the upper compartment of inserts alone or inserts with HIBCPP cells (Fig. 1a), and 24 h later the number of transmigrated cells was assessed by fluorescence measurements.

Between 0.14 and 0.22 % of neuroblastoma cells effectively transmigrated through the barrier and no significant difference could be seen between IMR32 and SH-SY5Y cell lines (Fig. 1b; 0.14 ± 0.02 and 0.22 ± 0.05 %, respectively). A larger proportion of neuroblastoma cells could reach the lower compartment in the absence of HIBCPP cells (Fig. 1b; 2.94 ± 0.10 % of IMR32 cells and 3.37 ± 0.33 % of SH-SY5Y), showing that the barrier formed by HIBCPP cells restricted cancer cell migration. No impairment of the barrier integrity could be seen after transmigration experiments, concerning either the TEER (Fig. 1c) or the permeability to LY (Fig. 1d), regardless of the neuroblastoma cell line used. This data suggests that the epithelial barrier function of HIBCPP layer was not impaired in presence of IMR32 and SH-SY5Y neuroblastoma cells.

When the barrier formed by HIBCPP cells was reversibly impaired by a pretreatment with the actin microfilament-disrupting agent Cytochalasin D, the transmigration rate of both neuroblastoma cell lines was increased compared to untreated filters (Additional file 1: Figure 1).

### The different steps of neuroblastoma cell transmigration through the BCSFB in vitro

**Fig. 2:**
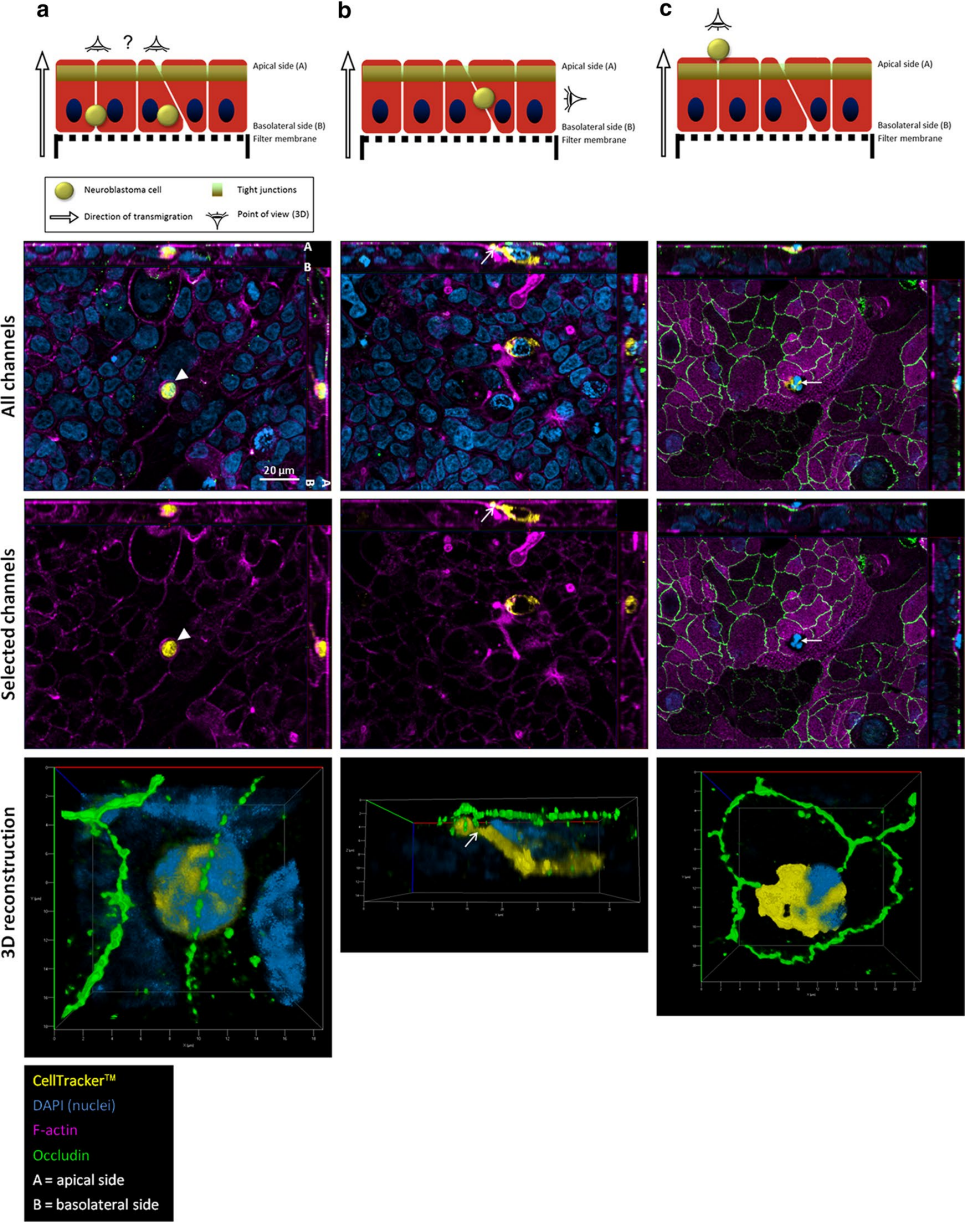
Fluorescent microscopy study of neuroblastoma cell transmigration through the BCSFB in vitro. Six hours after seeding SH-SY5Y neuroblastoma cells in the upper compartments of the filters, samples were fixed and stained for occludin (tight junction protein, in* green*), F-actin (cytoskeleton, in* pink*) and DAPI (nuclei, in *blue*). Neuroblastoma cells had previously been stained with a fixable CellTracker™ (in *yellow*). As represented for each set of pictures (*upper panels*), photographs show representative neuroblastoma cells at different stages of the transmigration process (during, **a**, **b**; and after, **c**) through the epithelial layer formed by HIBCPP cells on the* lower side* of the filters (two* middle panels*, *en face* Apotome® microscopy images showing all channels and selected channels, respectively;* lower panels*, 3D reconstructions from an apical—**a** and **c**—and a lateral point of view—**b**). Apical and basolateral sides of the barriers are indicated by ‘A’ and ‘B’, respectively. In **a** the *arrowheads* indicate the phalloidin-stained filamentous actin cytoskeleton surrounding the transmigrating neuroblastoma cell. In **b** the *arrows* point to a possible projection sent by the transmigrating cancer cell through tight junctions. In **c** *arrows* indicate the neuroblastoma cell having reached the apical surface of the epithelial barrier. *Scale bar* as indicated

To observe transmigrating neuroblastoma cells at different stages, experiments were stopped after 6 h by fixing the samples before immunocytochemical analysis and subsequent fluorescence microscopy observation (Fig. 2). For each set of pictures (a, b and c), upper panels indicate the localizations of cancer cells within the HIBCPP layer corresponding to the following pictures (middle and lower panels). Photographs show the layer formed by HIBCPP cells on the lower side of the filters (Fig. 2, two middle panels; apical and basolateral sides indicated by ‘A’ and ‘B’, respectively), and CellTracker™-stained neuroblastoma SH-SY5Y cells during (Fig. 2a, b) and after (Fig. 2c) trans-epithelial migration.

Figure 2a shows a neuroblastoma cell progressing within the epithelium, seemingly surrounded by the epithelial filamentous actin cytoskeleton (Fig. 2a, middle panels, arrowheads). The 3D reconstruction of a magnification from an apical point of view (Fig. 2a, lower panel) shows the rounded neuroblastoma cell progressing within the layer of epithelial cells, just below a tight junction, but the route taken by the cancer cell (paracellular or transcellular) cannot be definitely determined. In Fig. 2b, a neuroblastoma cell is also progressing through the barrier but exhibits an elongated shape. It is sending a possible projection (Fig. 2b, middle panels, arrows) to the apical side of the epithelium, through the tight junctions (Fig. 2b, lower panel; 3D reconstruction of a magnification from a lateral point of view). This data suggests that neuroblastoma cells can undergo paracellular trans-epithelial migration in order to cross the BCSFB. Finally, Fig. 2c represents a neuroblastoma cell having completed the transmigration process because it appears on the surface of the epithelial layer, expressing continuous tight junctions (Fig. 2c, middle panels). The 3D reconstruction from an apical point of view (Fig. 2c, lower panel) shows the cancer cell lying at the apical side of the barrier, with a continuous tight junction staining underneath the neuroblastoma cell (Additional file 2: Figure 2).

## Discussion

The improvement of treatment strategies has allowed for remissions in a large subset of high-risk neuroblastoma patients, but in parallel, an increasing number of children will later develop focal and metastatic relapse [7, 8]. Among the involved organs, the brain is one of the most common sites of isolated relapse in neuroblastoma patients [1, 7], and up to 16 % of patients develop CNS metastases [1, 9, 10]. The prognosis of these patients is still dismal despite extensive therapeutic efforts [1, 8, 11]. Therefore, understanding how neuroblastoma cells manage to reach the CNS despite its protective barriers is of utmost importance. The blood–brain barrier (BBB), located in brain microvascular endothelial cells, is thought to be the main entry gate for cancer cells into the CNS via the hematogenous route [12]. In neuroblastoma, patients not only develop parenchymal metastases, but also leptomeningeal and intraventricular lesions have been described [4]. In these patients, it might well be that the tumor cells have transmigrated through the BCSFB, after having crossed the fenestrated capillaries of the choroid plexus. However, rare studies have considered the BCSFB as a potential route for cancer cells into the brain [13], and therefore the significance of this phenomenon has not been explored until now.

Since in vitro models prove useful in deciphering cellular mechanisms involved in cancer cell-brain barrier interactions [14, 15], our aim was to use a human in vitro BCSFB model for studying the migration potential of neuroblastoma cells through the BCSFB. As already described [6], this human BCSFB model exhibits proper barrier features, as shown by its TEER and low permeability to LY. The present results are the first to demonstrate that two neuroblastoma cell lines (IMR-32 and SH-SY5Y) are able to cross a choroid plexus epithelial cell layer, representing the anatomical basis of the BCSFB. Transmigrating neuroblastoma cells could be detected at different stages of the process by fluorescence microscopy imaging and 3-dimensional reconstruction. No impairment in the barrier integrity could be observed after 6–24 h of contact and no focal destruction of the epithelial layer could be observed around the transmigrating neuroblastoma cells; therefore we assume an active transmigration process of neuroblastoma cells through the choroid plexus epithelial cell layer. From fluorescence microscopy imaging we cannot definitely distinguish a transcellular from a paracellular route. Although the significantly increased transmigration rate in case of BCSFB disruption by cytochalasin D argues for a paracellular way, electron microscopy imaging is warranted to gain further insights into this process. Previous studies have highlighted some alterations of endothelial biomechanical properties by cancer cells, involving actin cytoskeletal remodeling via Rho kinase signaling [16, 17]. The described rearrangement of endothelial actin filaments can promote the trans-endothelial migration of cancer cells by impacting tight junction assembly [17]. Similar processes might occur during the trans-epithelial migration of neuroblastoma cells herein described. Different molecules secreted by cancer cells are able to influence neighboring cells’ phenotype, in particular at the level of the cellular barriers with which they interact during the metastatic process [18]. Among these, transforming growth factor beta-1, which is secreted by neuroblastoma cells [19], can promote the extravasation process by inducing actin cytoskeleton reorganization [18, 20, 21]. Recently, microRNA-181c-containing extracellular vesicles secreted by breast cancer cell lines have been shown to induce BBB disruption via actin microfilament disorganization [22]. It cannot be excluded that neuroblastoma cells could cause cytoskeleton changes in choroid plexus epithelium by secreting such vesicles.

Brain metastases of extracranial malignancies are not restricted to parenchymal lesions. Metastases within the choroid plexus or the ventricles as well as leptomeningeal tumor spread have been described both in neuroblastoma [4] and various other malignancies in children and adults [23–25]. In these patients, the choroid plexus might have been an entry site of tumor cells to the CNS [23], with circulating cancer cells migrating from the lumen of the fenestrated choroidal capillaries to that of ventricles. Concerning adhesion to and transmigration across endothelial cells, cancer cells share many mechanisms with leukocytes including binding to selectins, integrins, and immunoglobulin-like cell adhesion molecules to invade various organs [18]. Since the epithelial cells of the BCSFB share the expression of many of these adhesion molecules with BBB endothelial cells [26, 27], and these cellular interactions are—at least in part—responsible for tumor cell adhesion to and transmigration through the BBB [28], we hypothesize that the BCSFB within the choroid plexus might represent a relevant site of tumor invasion to the CNS. However, most previous studies focused on the molecular and cellular interaction of tumor cells with the endothelial cells of the BBB [14, 15, 29], and therefore the role of the choroid plexus epithelial cells in the development of brain metastases still needs to be addressed. In this regard, human in vitro models of the BCSFB hold out the prospect of contributing significantly to our knowledge of molecular and cellular interactions between tumor cells and choroid plexus epithelial cells.

## Conclusions

We herein demonstrate for the first time that neuroblastoma cells are able to actively cross the BCSFB in a human in vitro model. These results indicate that the choroid plexus may be an underestimated site of tumor cell invasion to the CNS. To further elucidate underlying molecular mechanisms, in vitro models are powerful tools in deciphering cellular interactions of cancer cells and brain barriers in neuroblastoma, as well as in other malignancies that cause brain metastases.

## Methods

### *BCSFB* in vitro *model*

The BCSFB model is based on the cultivation of human choroid plexus papilloma cells (known as HIBCPP) on the lower surface of cell culture inserts, as already described [6] (Fig. 1a). In brief, HIBCPP were cultured in DMEM/HAM’s F12 1:1 (Ref. 31330, Gibco) supplemented with 15 % (v/v) heat-inactivated fetal calf serum (FCS), 4 mM l-Glutamine, 5 mg mL^−1^ insulin (Ref. I9278, Sigma), 100 U mL^−1^ penicillin and 100 mg mL^−1^ streptomycin (HIBCPP-medium with 15 % FCS). Cells were seeded on the lower surface of cell culture inserts (ThinCert™ Cell Culture Inserts for 24-well plates, pore diameter 3.0 µm; Ref. 662631, Greiner Bio-One, Frickenhausen, Germany) that were flipped over and placed in medium-flooded 12-well plates. Cells were fed the following day, and the filters were flipped over again and placed in a 24-well plate 2 days after seeding. Cell culture was continued in HIBCPP-medium containing 1 % FCS to increase TEER. When the TEER reached at least 250 Ω cm^2^, experiments could be launched.

### Cultivation of neuroblastoma cell lines

Human neuroblastoma cell lines SH-SY5Y and IMR32 were purchased from the European Collection of Cell Cultures (Cat n° 94030304 and 86041809, respectively). They were cultured in vitro under standard culture conditions (37 °C, 5 % CO_2_ under humidified atmosphere) in RPMI 1640 medium (Ref. F1215, Biochrom AG) supplemented with 10 % FCS, 4 mM l-Glutamine (Ref. 25030, Gibco), 1 % (v/v) non-essential amino acids (Ref. 11140, Gibco), 100 U mL^−1^ penicillin and 100 µg.mL^−1^ streptomycin. Medium was renewed every 2–3 days. Before reaching confluence, cells were routinely harvested for passaging using 0.25 % trypsin—0.02 % EDTA (ethylenediaminetetraacetic acid) solution (Ref. 25200, Gibco).

### Trans-epithelial electrical resistance (TEER) measurements

The TEER of HIBCPP cultures grown on inserts was measured using an epithelial tissue Volt-Ohm Meter using the STX-2 electrode system (Millicell ERS-2, Millipore, Schwalbach, Germany), before and after each transmigration experiment. The values measured for empty filters (or for filters only containing neuroblastoma cells in the case of transmigration experiment) were subtracted from the overall TEER values, in order to consider the resistance of the HIBCPP layer itself.

### Transmigration assays

Transmigration experiments were performed using the same medium in the upper and lower compartments (serum-free medium containing 0.5 % (w/v) Bovine Serum Albumin, BSA).

When experiments were carried out to determine the amount of transmigrated cells after 24 h, neuroblastoma cells were harvested, dissociated and labelled with the cell-permeant fluorescent dye BCECF-AM (2′,7′-Bis-(2-Carboxyethyl)-5-(and-6)-Carboxyfluorescein, Acetoxymethyl Ester), before being added to the upper compartment of the inserts, which represents the blood compartment in this inverted BCSFB model (Fig. 1a; 200,000 neuroblastoma cells added per insert). In parallel, the same number of neuroblastoma cells was added to the upper compartments of inserts alone, to assess the migration rate in the absence of any cellular barrier. After 24 h, the fluid in the lower compartments was centrifuged (5 min at 300×*g*) to pellet neuroblastoma cells located at the bottom of the wells. These cells were lysed using 1 % (w/v) Triton X-100 in PBS and their amount determined by fluorescence measurement with a Tecan 200 M Infinite Multiwell reader.

For the experiments that aimed at observing the transmigration process using immunocytochemistry (see next paragraph), neuroblastoma cells were instead loaded with Cell Tracker Green CMFDA (fixable dye) according to manufacturer’s instruction (Ref. C7025, Invitrogen). In this case, the experiment was stopped after 6–8 h.

### Immunocytochemistry

At the end of the transmigration experiment, cells were fixed with 4 % formaldehyde, diluted in PBS-CMF (phosphate-buffered saline, calcium- and magnesium-free), for 10 min and rinsed twice with PBS-CMF before cutting the membranes. They were subsequently permeabilized with 0.5 % (w/v) Triton X-100 diluted in PBS-CMF containing 1 % (w/v) BSA (PBS-CMF-1 %BSA) for 1 h. After rinsing three times with PBS-CMF-1 %BSA, samples were blocked with PBS-CMF-1 % BSA for 30 min and were then incubated with the primary antibody overnight at 4 °C (rabbit anti-occludin, Ref. 71-1500, Invitrogen; dilution 1/200 in PBS-CMF-1 %BSA).

The following day, samples were rinsed three times with PBS-CMF-1 %BSA, and incubated with the secondary antibody for 1 h at RT (chicken anti-rabbit IgG 594, Ref. A21442, Invitrogen, diluted 1/500 in PBS-CMF-1 %BSA).

After two rinsing steps with PBS-CMF-1 %BSA, nuclei were stained with 4′,6-Diamidino-2-phenylindole dihydrochloride (DAPI, dilution 1.5:50000 in PBS-CMF-1 %BSA) solution and the actin cytoskeleton with phalloidin (Phalloidin Alexa fluor^®^ 660, Invitrogen; dilution 1:250 in PBS-CMF-1 %BSA,) for 5 min. Finally, the preparations were rinsed three times with PBS-CMF and mounted under coverslips with Prolong Antifade reagent (Invitrogen).

Images were acquired and processed with Zeiss Apotome and Axiovision software (Carl Zeiss, Jena, Germany) using a 63x/1.4 objective lens. This system provides an optical slice view reconstructed from fluorescent samples (structured illumination microscopy).

### Permeability of the barrier to a low-molecular-weight integrity marker

To assess the integrity of the BCSFB in vitro, its permeability to Lucifer Yellow (LY, 457 Da, Ref. L0259, Sigma-Aldrich) was measured in control filters and after each transmigration experiment. The quantity of LY having crossed the BCSFB in vitro was measured in the lower compartment after 1 h, using a microplate reader (Tecan Infinite M200 Multiwell reader, Tecan, Switzerland). For permeability coefficient (Pe) calculations, the method from Cecchelli et al. [30] was used.

### Statistical analyses

The results were expressed as mean ± SEM from three independent experiments, each performed in triplicates. Statistical significance was assessed by unpaired t-tests. A *p* value <0.05 was considered as significant. All statistical analyses were performed using GraphPad Prism 5.0 for Windows (GraphPad Software, San Diego, California, USA).

## Additional files

**Additional file 1: Figure 1.** Effect of a pretreatment of the HIBCPP layer with the actin microfilament-disrupting agent Cytochalasin D on the transmigration rate of IMR32 and SH-SY5Y neuroblastoma cell lines (**a**) and barrier integrity, as assessed by TEER (**b**) and dextran flux (**c**) measurements. Transmigration experiments were performed and transmigration rates were determined as described in Materials and Methods section (**a**). Before transmigration experiments, filters with HIBCPPs were incubated for 75 min with 1 µg.ml^−1^ Cytochalasin D (Sigma) diluted in serum-free medium containing 0.5 % BSA (‘+ cytochalasin D’ condition). In parallel, control filters were incubated with serum-free medium containing 0.5 % BSA (‘- cytochalasin D’ condition). The TEER was measured before the treatment and after the treatment to confirm break-down of the barrier properties (**b**, ‘before’ and ‘cyto D’ conditions). All filters were then placed in new wells containing medium without Cytochalasin D, the transmigration experiment was launched. 5 µl Dextran-TexasRed (MW: 3000 Da, Life Technologies) were added to the upper compartment of the inserts together with IMR32 or SH-SY5Y cells, in order to monitor permeability of HIBCPPs treated with and without cytochalasin D during the experiment (**c**). After 4 h of transmigration, the TEER was measured again (**b**, condition ‘after’). TEER values increased again and the experiment was stopped. The fluid in the lower compartments was collected for determination of the amount of Dextran having crossed the barrier during the experiment by fluorescence measurement using a Tecan 200 M Infinite Multiwell reader (**c**). All results were expressed as mean ± SD from two independent experiments, each performed in triplicates. Statistical significance was assessed by unpaired t-tests. A p-value < 0.05 was considered as significant. Statistical analyses were performed using GraphPad Prism 5.0 for Windows (GraphPad Software, San Diego, California, USA).

**Additional file 2: Figure 2.** Three-dimensional reconstruction from a basolateral point of view of Figure 2c. It shows that the transmigrated cancer cell is lying over a continuous tight junction, at the apical side of the barrier. Scale bar as indicated.

## Abbreviations

BBB: blood–brain barrier
BCECF-AM: 2′,7′-Bis-(2-CarboxyEthyl)-5-(and-6)-CarboxyFluorescein, AcetoxyMethyl ester
BCSFB: blood-cerebrospinal fluid barrier
BSA: bovine serum albumin
CNS: central nervous system
CSF: cerebrospinal fluid
DAPI: 4′,6-Diamidino-2-phenylindole dihydrochloride
EDTA: ethylenediaminetetraacetic acid
DMEM: Dulbecco’s modified Eagle’s medium
FCS: fetal calf serum
HIBCPP: human choroid plexus papilloma cells
LY: Lucifer Yellow
PBS: phosphate-buffered saline
PBS-CMF: phosphate-buffered saline, calcium- and magnesium-free
TEER: trans-epithelial electrical resistance

## References

[1] Matthay KK, Brisse H, Couanet D, Couturier J, Bénard J, Mosseri V, Edeline V, Lumbroso J, Valteau-Couanet D, Michon J (2003). Central nervous system metastases in neuroblastoma: radiologic, clinical, and biologic features in 23 patients. Cancer.

[2] Wiens AL, Hattab EM (2014). The pathological spectrum of solid CNS metastases in the pediatric population. J Neurosurg Pediatr.

[3] Strazielle N, Ghersi-Egea JF (2013). Physiology of blood-brain interfaces in relation to brain disposition of small compounds and macromolecules. Mol Pharm.

[4] Nabavizadeh SA, Feygin T, Harding BN, Bilaniuk LT, Zimmerman RA, Vossough A (2014). Imaging findings of patients with metastatic neuroblastoma to the brain. Acad Radiol.

[5] Codreanu I, Dasanu CA, Zhuang H (2014). Neuroblastoma with a Solitary Intraventricular Brain Metastasis Visualized on I-123 MIBG Scan. J Neuroimaging.

[6] Schwerk C, Papandreou T, Schuhmann D, Nickol L, Borkowski J, Steinmann U, Quednau N, Stump C, Weiss C, Berger J, Wolburg H, Claus H, Vogel U, Ishikawa H, Tenenbaum T, Schroten H (2012). Polar invasion and translocation of neisseria meningitidis and streptococcus suis in a novel human model of the blood-cerebrospinal fluid barrier. PLoS One.

[7] Cheung NKV, Cheung IY, Kushner BH, Ostrovnaya I, Chamberlain E, Kramer K, Modak S (2012). Murine anti-GD2 monoclonal antibody 3F8 combined with granulocyte- macrophage colony-stimulating factor and 13-cis-retinoic acid in high-risk patients with stage 4 neuroblastoma in first remission. J Clin Oncol.

[8] Kramer K, Kushner B, Heller G, Cheung NKV (2001). Neuroblastoma metastatic to the central nervous system the memorial sloan-kettering cancer center experience and a literature review. Cancer.

[9] Blatt J, Fitz C, Mirro J (1997). Recognition of central nervous system metastases in children with metastatic primary extracranial neuroblastoma. Pediatr Hematol.

[10] DuBois SG, Kalika Y, Lukens JN, Brodeur GM, Seeger RC, Atkinson JB, Haase GM, Black CT, Perez C, Shimada H, Gerbing R, Stram DO, Matthay KK (1999). Metastatic sites in stage IV and IVS neuroblastoma correlate with age, tumor biology, and survival. J Pediatr Hematol Oncol.

[11] Kellie SJ, Hayes FA, Bowman L, Kovnar EH, Langston J, Jenkins JJ, Pao WJ, Ducos R, Green AA (1991). Primary extracranial neuroblastoma with central nervous system metastases characterization by clinicopathologic findings and neuroimaging. Cancer.

[12] Preusser M, Capper D, Ilhan-Mutlu A, Berghoff AS, Birner P, Bartsch R, Marosi C, Zielinski C, Mehta MP, Winkler F, Wick W, Von Deimling A (2012). Brain metastases: Pathobiology and emerging targeted therapies. Acta Neuropathol..

[13] Hochman J, Assaf N, Deckert-Schlüter M, Wiestler OD (2001). Pe’er J: Entry routes of malignant lymphoma into the brain and eyes in a mouse model. Cancer Res.

[14] Choi YP, Lee JH, Gao MQ, Kim BG, Kang S, Kim SH, Cho NH (2014). Cancer-associated fibroblast promote transmigration through endothelial brain cells in three-dimensional in vitro models. Int J Cancer.

[15] Fazakas C, Wilhelm I, Nagyoszi P, Farkas AE, Haskó J, Molnár J, Bauer H, Bauer HC, Ayaydin F, Dung NTK, Siklós L, Krizbai IA (2011). Transmigration of melanoma cells through the blood-brain barrier: role of endothelial tight junctions and melanoma-released serine proteases. PLoS One.

[16] Mierke CT (2011). Cancer cells regulate biomechanical properties of human microvascular endothelial cells. J Biol Chem.

[17] Li B, Zhao W-D, Tan Z-M, Fang W-G, Zhu L, Chen Y-H (2006). Involvement of Rho/ROCK signalling in small cell lung cancer migration through human brain microvascular endothelial cells. FEBS Lett.

[18] Reymond N, D’Água BB, Ridley AJ (2013). Crossing the endothelial barrier during metastasis. Nat Rev Cancer.

[19] Castriconi R, Dondero A, Bellora F, Moretta L, Castellano A, Locatelli F, Corrias MV, Moretta A, Bottino C (2013). Neuroblastoma-derived TGF-β1 modulates the chemokine receptor repertoire of human resting NK cells. J Immunol.

[20] Lu Q, Harrington EO, Jackson H, Morin N, Shannon C, Rounds S (2006). Transforming growth factor-beta1-induced endothelial barrier dysfunction involves Smad2-dependent p38 activation and subsequent RhoA activation. J Appl Physiol.

[21] Antonov AS, Antonova GN, Fujii M, ten Dijke P, Handa V, Catravas JD, Verin AD (2012). Regulation of endothelial barrier function by TGF-beta type I receptor ALK5: potential role of contractile mechanisms and heat shock protein 90. J Cell Physiol.

[22] Tominaga N, Kosaka N, Ono M, Katsuda T, Yoshioka Y, Tamura K, Lötvall J, Nakagama H, Ochiya T (2015). Brain metastatic cancer cells release microRNA-181c-containing extracellular vesicles capable of destructing blood–brain barrier. Nat Commun..

[23] Shapira Y, Hadelsberg UP, Kanner AA, Ram Z, Roth J (2014). The ventricular system and choroid plexus as a primary site for renal cell carcinoma metastasis. Acta Neurochir (Wien).

[24] Suki D (2014). Khoury Abdulla R, Ding M, Khatua S, Sawaya R: Brain metastases in patients diagnosed with a solid primary cancer during childhood: experience from a single referral cancer center. J Neurosurg Pediatr.

[25] Siomin V, Lin JL, Marko NF, Barnett GH, Toms SA, Chao ST, Angelov L, Vogelbaum MA, Navaratne K, Suh JH, Weil RJ (2014). Stereotactic radiosurgical treatment of brain metastases to the choroid plexus. Int J Radiat Oncol Biol Phys.

[26] Engelhardt B, Sorokin L (2009). The blood-brain and the blood-cerebrospinal fluid barriers: Function and dysfunction. Semin Immunopathol.

[27] Redzic Z (2011). Molecular biology of the blood-brain and the blood-cerebrospinal fluid barriers: similarities and differences. Fluids Barriers CNS.

[28] Martinez N, Boire A, DeAngelis LM (2013). Molecular interactions in the development of brain metastases. Int J Mol Sci..

[29] Wilhelm I, Molnár J, Fazakas C, Haskó J, Krizbai IA (2013). Role of the blood-brain barrier in the formation of brain metastases. Int J Mol Sci..

[30] Cecchelli R, Dehouck B, Descamps L, Fenart L, Buée-Scherrer V, Duhem C, Lundquist S, Rentfel M, Torpier G, Dehouck MP (1999). In vitro model for evaluating drug transport across the blood-brain barrier. Adv Drug Deliv Rev..

