# Supplementary figures and images for: The choroid plexus may be an underestimated site of tumor invasion to the brain: an in vitro study using neuroblastoma cell lines
Source: Cancer Cell Int. 2015 Oct 24;15:102. doi: 10.1186/s12935-015-0257-2 (PMC4619509; doi:10.1186/s12935-015-0257-2)

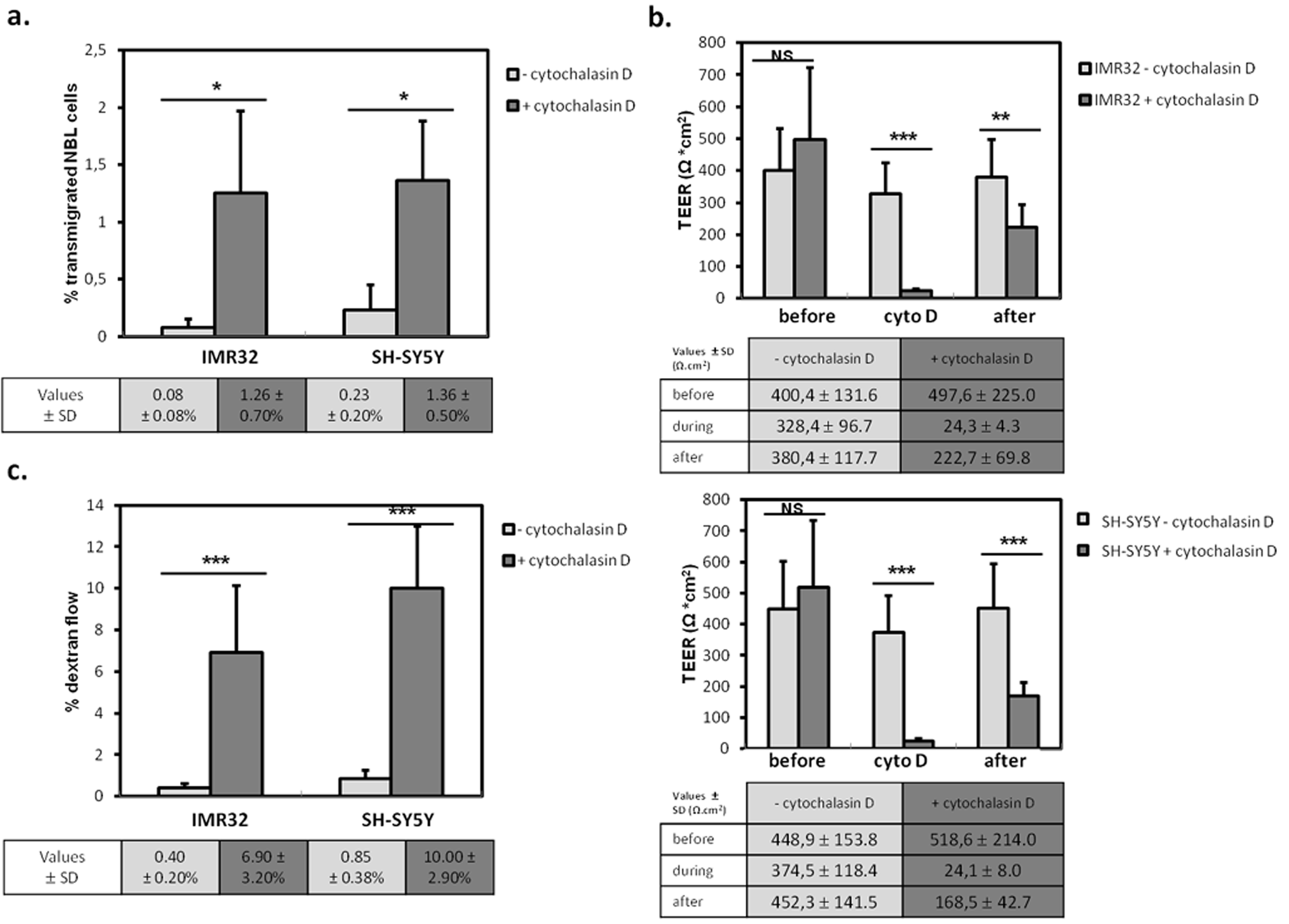

Supplement: Supplementary file 1 — Additional file 1: Figure 1. Effect of a pretreatment of the HIBCPP layer with the actin microfilament-disrupting agent Cytochalasin D on the transmigration rate of IMR32 and SH-SY5Y neuroblastoma cell lines (a) and barrier integrity, as assessed by TEER (b) and dextran flux (c) measurements. Transmigration experiments were performed and transmigration rates were determined as described in Materials and Methods section (a). Before transmigration experiments, filters with HIBCPPs were incubated for 75 min with 1 µg.ml−1 Cytochalasin D (Sigma) diluted in serum-free medium containing 0.5 % BSA (‘+ cytochalasin D’ condition). In parallel, control filters were incubated with serum-free medium containing 0.5 % BSA (‘- cytochalasin D’ condition). The TEER was measured before the treatment and after the treatment to confirm break-down of the barrier properties (b, ‘before’ and ‘cyto D’ conditions). All filters were then placed in new wells containing medium without Cytochalasin D, the transmigration experiment was launched. 5 µl Dextran-TexasRed (MW: 3000 Da, Life Technologies) were added to the upper compartment of the inserts together with IMR32 or SH-SY5Y cells, in order to monitor permeability of HIBCPPs treated with and without cytochalasin D during the experiment (c). After 4 h of transmigration, the TEER was measured again (b, condition ‘after’). TEER values increased again and the experiment was stopped. The fluid in the lower compartments was collected for determination of the amount of Dextran having crossed the barrier during the experiment by fluorescence measurement using a Tecan 200 M Infinite Multiwell reader (c). All results were expressed as mean ± SD from two independent experiments, each performed in triplicates. Statistical significance was assessed by unpaired t-tests. A p-value < 0.05 was considered as significant. Statistical analyses were performed using GraphPad Prism 5.0 for Windows (GraphPad Software, San Diego, California, USA). [file 12935_2015_257_MOESM1_ESM.tif]

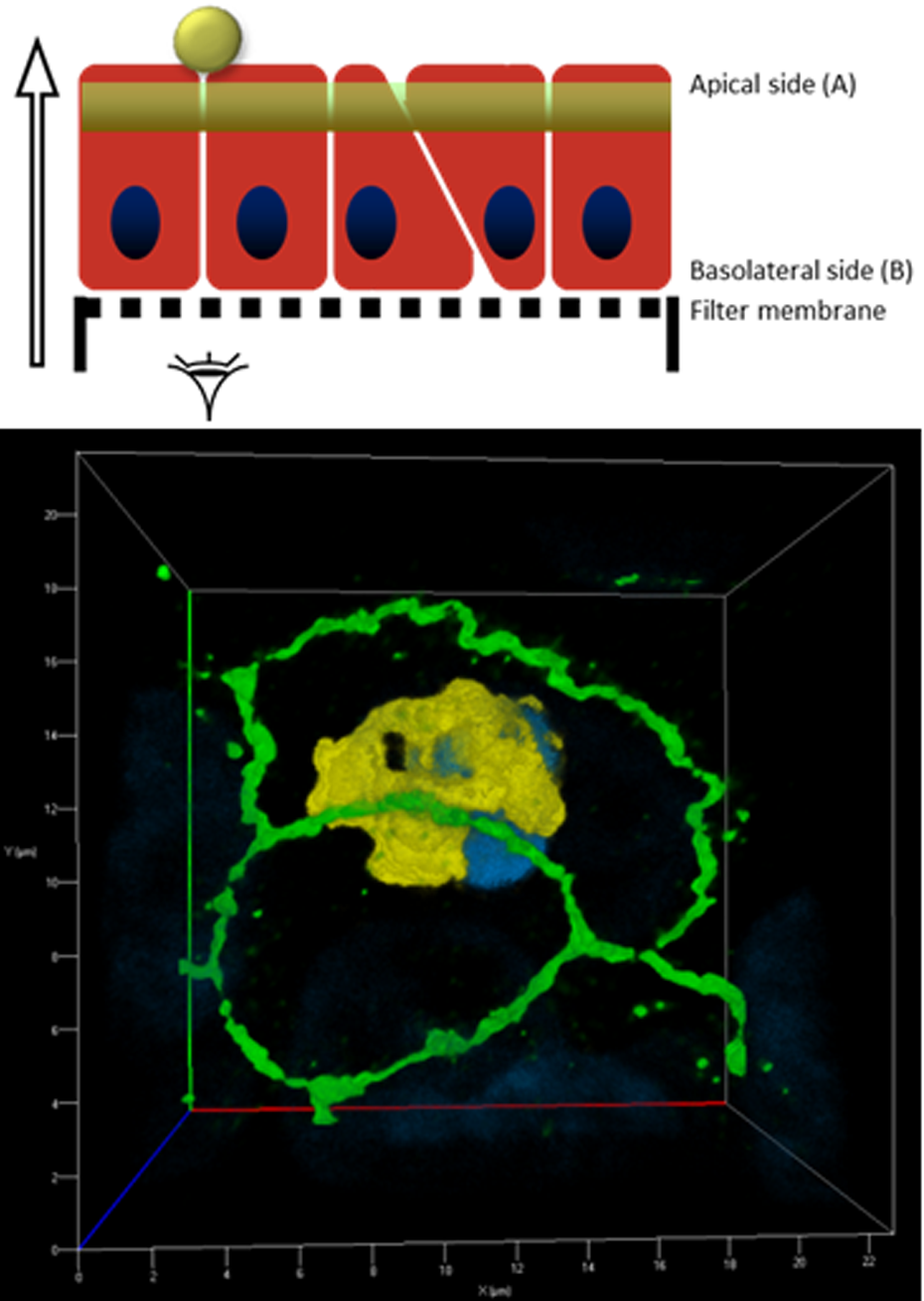

Supplement: Supplementary file 2 — Additional file 2: Figure 2. Three-dimensional reconstruction from a basolateral point of view of Figure 2c. It shows that the transmigrated cancer cell is lying over a continuous tight junction, at the apical side of the barrier. Scale bar as indicated. [file 12935_2015_257_MOESM2_ESM.tif]
